# Healthfulness Assessment of Recipes Shared on Pinterest: Natural Language Processing and Content Analysis

**DOI:** 10.2196/25757

**Published:** 2021-04-20

**Authors:** Xiaolu Cheng, Shuo-Yu Lin, Kevin Wang, Y Alicia Hong, Xiaoquan Zhao, Dustin Gress, Janusz Wojtusiak, Lawrence J Cheskin, Hong Xue

**Affiliations:** 1 Department of Health Administration and Policy College of Health and Human Services George Mason University Fairfax, VA United States; 2 Department of Communication College of Humanities and Social Sciences George Mason University Fairfax, VA United States; 3 Department of Nutrition and Food Studies College of Health and Human Services George Mason University Fairfax, VA United States

**Keywords:** healthfulness assessment, recipes on Pinterest, social networks, natural language processing

## Abstract

**Background:**

Although Pinterest has become a popular platform for distributing influential information that shapes users’ behaviors, the role of recipes pinned on Pinterest in these behaviors is not well understood.

**Objective:**

This study aims to explore the patterns of food ingredients and the nutritional content of recipes posted on Pinterest and to examine the factors associated with recipes that engage more users.

**Methods:**

Data were collected from Pinterest between June 28 and July 12, 2020 (207 recipes and 2818 comments). All samples were collected via 2 new user accounts with no search history. A codebook was developed with a raw agreement rate of 0.97 across all variables. Content analysis and natural language processing sentiment analysis techniques were employed.

**Results:**

Recipes using seafood or vegetables as the main ingredient had, on average, fewer calories and less sodium, sugar, and cholesterol than meat- or poultry-based recipes. For recipes using meat as the main ingredient, more than half of the energy was obtained from fat (277/490, 56.6%). Although the most followed pinners tended to post recipes containing more poultry or seafood and less meat, recipes with higher fat content or providing more calories per serving were more popular, having more shared photos or videos and comments. The natural language processing–based sentiment analysis suggested that Pinterest users weighted *taste* more heavily than *complexity* (225/2818, 8.0%) and *health* (84/2828, 2.9%).

**Conclusions:**

Although popular pinners tended to post recipes with more seafood or poultry or vegetables and less meat, recipes with higher fat and sugar content were more user-engaging, with more photo or video shares and comments. Data on Pinterest behaviors can inform the development and implementation of nutrition health interventions to promote healthy recipe sharing on social media platforms.

## Introduction

### Background

Healthy eating patterns and their effect on disease prevention have been demonstrated robustly across the scientific literature [1-9]. The US government has dedicated enormous resources to improve Americans’ eating patterns through programs such as the National School Lunch program and the Special Supplemental Nutrition Program for Women, Infants, and Children. In 2015, the Dietary Guidelines Advisory Committee published the 2015-2020 Dietary Guidelines for Americans, providing guidance for choosing a healthy diet. Despite such efforts, between 2003 and 2016, although the intake of sugar by Americans decreased by 4.8 teaspoons per day, no appreciable changes occurred in the intake of vegetables, total meat, poultry, and seafood [10]. Although from 2003-2004 to 2015-2016, Americans increased whole grain consumption, the mean intake of grains, vegetables, and dairy continued to be lower than the Dietary Guidelines recommendations [10]. Only 42% of the US population met dietary recommendations between 2013 and 2014, and less than half of the older adults met the recommendations between 2013 and 2016 [11,12].

Social media has become a new and efficient way to distribute and consume influential information that shapes people’s dietary behaviors [13,14]. Several internet-based intervention programs have been implemented to enhance individuals’ knowledge of healthy eating [15,16]. With the growing popularity of social media, there is an urgent need to assess the contents of healthy food and nutrition information on social media and their associations with user engagement among both posters and information seekers.

Pinterest, launched in 2010, is a unique social media platform where users can save images (*pins*) and upload it to the board (a collection of pins from different users) [17]. It has also become a popular social media site for users to share recipes. According to a survey conducted by the Pew Research Center in 2018, 28% of US adults reported that they had used Pinterest [18], and over 60% of active users made a new recipe inspired by Pinterest in 2015 [19]. Pinterest provides us with a platform and unprecedented opportunities to study the effect of social media on dietary behaviors and, consequently, public health [20]. This study aims to examine the patterns in which nutrition information on recipes is received and shared among Pinterest users and identify the key elements of recipes that influence the perceptions and preferences of Pinterest users. Our findings will shed light on future social media–based dietary intervention program design and implementation.

### Objectives

To the best of our knowledge, no prior study has evaluated the recipe content on Pinterest. This study provides a first glimpse of this domain to advance the understanding of the relationship between social media use and dietary behavior. We aim to achieve the following 2 goals. First, we aim to examine the patterns of food ingredients and nutrients prescribed by recipes posted on Pinterest. Second, by employing both traditional content analysis and a natural language processing (NLP) technique, we sought to understand the factors that distinguish the most popular recipes among users.

## Methods

### Data Collection

Data were collected between June 28 and July 12, 2020. Although there is no “rule of thumb” on how long the data collection should persist, we adapted a proper time frame based on previous literature that specifically focused on Pinterest [21,22]. All samples were collected using the Pinterest search engine by 2 new user accounts with no search history, no posts and boards or pins, 0 followers, and 0 following. The keywords *recipe*, *breakfast*, *lunch*, and *dinner* were used to identify samples on Pinterest. Pins with recipes containing all the required information were selected by scrolling down the search results page for each keyword. This approach was developed based on previous studies on Pinterest content [21,22]. Pins that were duplicates or missing any of the following information were excluded: eating occasion, cooking method, cooking time, ingredients, and nutrition information. A total of 207 collected pins or boards that met our criteria and all comments (2818 comments) under the 207 recipes were included in the analysis. A codebook was developed in an Excel spreadsheet (Microsoft Corp) to document the URL, time of data collection, comments, number of replied photos and videos, poster’s number of followers, eating occasion, cooking method, cooking time, ingredients, and nutrition information.

### Data Analysis

For the content analysis, following the 2015-2020 United States Department of Agriculture Dietary Guidelines, food ingredients were classified as dark green vegetables, red and orange vegetables, legumes (beans and peas), starchy vegetables, other vegetables, fruits, seafood, meats, poultry, eggs, nuts or seeds or soy products, dairy, oil, and butter [23]. The recipes were then categorized into 4 types based on their primary ingredients: meat, poultry, seafood, and vegetable recipes. Recipes that only contained meat, poultry, or seafood were further categorized according to whether they contained any vegetables. All measurement units were converted into grams per serving. The variables measured at the nutrient level included total energy (calories per serving); sodium (mg per serving); and, in grams per serving, fat, protein, carbohydrate, fiber, sugar, and cholesterol. Each pinner’s number of followers was classified based on a tertile distribution. Overall, 2 coders independently analyzed all study samples and performed cross-checks to ensure intercoder reliability. The raw agreement rate was 0.97 for all variables.

For the comment analysis, 3 keyword dictionaries were created with keywords related to health (eg, *health*, *healthy*, *calorie*, and *cholesterol*), taste (eg, *yummy*, *delicious*, *tasty*, and *creamy*), and the complexity of the recipe (eg, *quick*, *easy*, *sample*, and *difficult*). Keyword searching was applied to assess sentiment of comments posted by Pinterest users.

**Figure 1:**
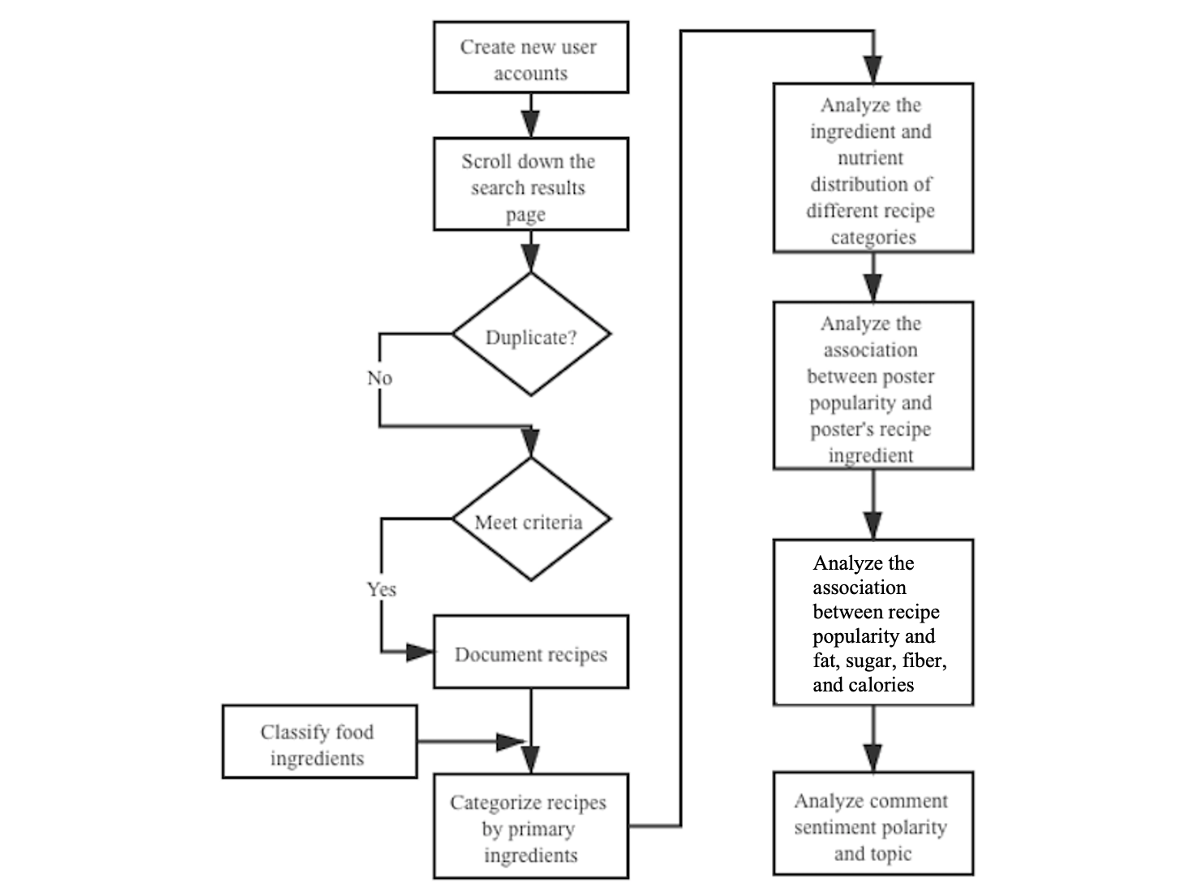
Process of data collection and analysis.

Descriptive analyses were performed for each type of food ingredient and their corresponding nutrient content. In addition, the popularity of recipe ingredients was assessed by the number of recoded followers (presented in tertiles). The level of engagement for each recipe was also evaluated by categorizing comments and shared photos or videos into tertiles, with regard to the fat, sugar, and fiber content of the recipes. Comments and shared photos or videos were chosen as indicators of engagement based on prior literature that suggested that, in the context of Pinterest, the number of *likes* on each pin indicates relatively low engagement (users simply acknowledge or agree with content), whereas the number of comments indicates medium engagement (users created and shared such content) [24]. High engagement is indicated by actual offline participation and can be captured by users’ shared photo or videos (images of what they made based on the same recipes) [24]. To process and analyze natural language data from the comments, both keyword search and sentiment analysis technology were applied. The sentiment analysis method we used was VADER (valence aware dictionary and sentiment reasoner). VADER performs sentiment analysis on textual data to determine whether the sentiment of a text is positive, negative, or neutral. VADER is a lexicon- and rule-based sentiment analysis method. It was developed specifically to analyze the sentiment of English text in microblog-like social media [25]. VADER requires no training data and provides high-speed analysis [26]. VADER helped us categorize all comments as being positive, neutral, or negative by polarity, that is, the representation of sentiment. All statistical analyses were conducted using STATA 15.1 (StataCorp LP) and Python 3.6 (Python Software Foundation). Figure 1 shows the entire data collection and analysis process.

## Results

### Summary Statistics

Table 1 describes the quantity of food ingredients and nutrients served by meat, poultry, seafood, and vegetable recipes. Although not statistically significant, 7 patterns were identified in the data. First, we found that meat, poultry, and seafood weighed more in recipes that only served these main dishes than those that also contained vegetables. For example, 124.79 g meat was served in meat-only recipes compared with 100.65 g in meat recipes that also contained vegetables. Second, recipes containing vegetables provided more total energy than recipes served without vegetables. For instance, poultry recipes that contained vegetables provided an average of 442.79 calories per serving compared with 433.31 calories provided by poultry-only recipes. Third, seafood recipes (329.38 calories per serving) or vegetable recipes (293.67 calories per serving) provided lower total energy content compared with meat- or poultry-based recipes (430-490 calories per serving). Fourth, 48.6% (230/473) and 56.5% (277/490) of the total energy in meat-only and meat-with-vegetable recipes came from fat, higher than the other types of recipes. Fifth, meat recipes, in general, contained higher fat (25.62-30.13 g per serving) than poultry- and seafood-based recipes (16.79-22.35 g per serving). Sixth, seafood recipes tended to contain less sodium and sugar than meat and poultry recipes. Seventh, 38.2% (112/293) of total energy in vegetable-based recipes came from carbohydrate, with less sodium (483.63 mg per serving) and cholesterol (47.65 g per serving), compared with other types of recipes.

**Table 1:** The ingredient and nutrient distributions of meat, poultry, seafood, and vegetable recipes.

Recipes			Meat^a^					Poultry^a^					Seafood^a^					Vegetable^a,b^ with eggs^c^ (n=59), mean (SD)
			Meat only (n=25), mean (SD)		Meat with vegetable^c^ (n=45), mean (SD)		Poultry only (n=35), mean (SD)			Poultry with vegetable^c^ (n=76), mean (SD)		Seafood only (n=6), mean (SD)			Seafood with vegetable^c^ (n=13), mean (SD)			
**Food ingredients (g per serving)^d^**																		
	Dark vegetable	N/A^e^		75.6 (0)		N/A			49.6 (14.1)		N/A			29.5 (15.4)		113.3 (0)		
	Red and other vegetable	N/A		90.6 (189.8)		N/A			29.5 (21.3)		N/A			N/A		72.5 (88.6)		
	Legumes and beans	N/A		42.5 (0)		N/A			81.5 (35.1)		N/A			N/A		70.8 (0)		
	Starchy vegetable	N/A		132.3 (26.8)		N/A			81.5 (35.1)		N/A			N/A		144.7 (129.2)		
	Meat	124.8 (128.5)		100.7 (98.2)		N/A			N/A		N/A			N/A		N/A		
	Poultry	N/A		N/A		106.7 (94.5)			100.4 (88.9)		N/A			N/A		N/A		
	Seafood	N/A		N/A		N/A			N/A		122.8 (66.2)			118.4 (73.0)		N/A		
	Eggs	51.4 (111.0)		51.4 (111.0)		14.5 (5.7)			16.6 (6.3)		N/A			N/A		28.3 (23.4)		
Total energy (calories per serving)			490.9 (280.1)		473.8 (246.8)		433.3 (225.5)			442.7 (185.7)		320.6 (126.5)			329.3 (178.5)		293.6 (131.6)	
**Nutrients (g per serving)**																		
	Fat	30.1 (17.9)		25.6 (17.4)		22.3 (13.8)			20.9 (13.0)		18.5 (9.6)			16.7 (13.7)		14.8 (9.7)		
	Percentage of energy from fat (%)^f^	56.6		48.5		46.7			41.8		50.5			40.4		44.8		
	Protein	26.4 (11.9)		28.5 (19.2)		32.6 (19.5)			36.8 (50.2)		23.5 (14.9)			27.1 (16.6)		12.3 (10.0)		
	Percentage of energy from protein (%)^f^	24.2		25.2		30.9			34.9		30.7			33.1		16.4		
	Carbohydrates	24.7 (17.7)		27.3 (20.0)		20.9 (24.2)			27.1 (24.6)		12.6 (17.1)			16.3 (17.1)		29.1 (24.1)		
	Percentage of energy from carbohydrates (%)^f^	22.7		25.6		17.9			23.8		14.5			24.9		38.3		
	Fiber	2.2 (2.)		3.8 (7.6)		1.3 (1.2)			2.9 (3.6)		0.8 (0.7)			1.7 (2.1)		4.0 (5.1)		
	Sodium (mg per serving)	861.2 (655.9)		775.6 (683.3)		911.0 (510.6)			767.0 (559.5)		489.8 (431.6)			777.3 (659.1)		483.6 (389.1)		
	Sugar	6.5 (12.9)		6.7 (11.6)		5.7 (8.0)			6.6 (7.9)		1.3 (1.0)			3.5 (4.2)		4.73 (7.81)		
	Cholesterol	114.6 (135.5)		97.2 (113.1)		126.5 (71.4)			109.3 (69.8)		98.5 (100.6)			152.9 (177.2)		47.6 (66.5)		

^a^Recipes that included a main dish only and those that included a main dish served with vegetables were mutually exclusive. For example, a meat-only recipe was defined as a recipe that only included meat, whereas recipes that included both meat and vegetables were listed in the meat with vegetable category.

^b^No recipes were purely vegan; therefore, we reported ovo-lacto recipes.

^c^The calculation of sample average used only complete data, that is, some of the denominators were smaller than 45 and did not have standard errors.

^d^The food ingredients were categorized based on the guidelines of the United States Department of Agriculture.

^e^N/A: not applicable.

^f^The sum of column percentages of each recipe class may exceed 100% because each value was calculated separately as the percentage of energy from specific nutrients divided by the total energy provided in a recipe class.

### Relationship Between Popularity and Ingredients of Shared Recipes

The bar charts in Multimedia Appendix 1 describe the relationship between the pinner’s popularity and the ingredients of their shared recipes. Popularity was measured as the number of followers per pinner, stratified by tertile. Pinners in third tertile had more followers. Although not statistically significant, 3 patterns emerged. First, as the number of followers increased, the amount of meat served in the recipe decreased. Second, recipes with greater followings contained more poultry and seafood but fewer red and other vegetables. Third, starchy vegetables were distributed similarly, regardless of the number of followers.

### Relationship Between Photo or Video and Comments Sharing and Recipe Features

Multimedia Appendix 2 presents the mean number of shared photos or videos and comments under the recipes according to the absolute amount of fat per serving in the recipes, classified in tertiles. Recipes in the third tertile had the highest fat content. Data showed that more photos or videos and comments were shared as the absolute amount of fat per serving increased. Although not statistically significant, among the 172 recipes with complete information, the average number of shared photos or videos and comments increased from 25 to 35 between the first and third tertiles, suggesting that recipes with higher fat content, such as creamy garlic butter chicken, were more popular among Pinterest users.

Multimedia Appendix 2 also presents the mean number of shared photos or videos and comments under recipes based on the recipes’ sugar content. Among 166 recipes that had complete sugar information, the mean number of photos or videos and comments were distributed as an inverse *U* shape. Although not statistically significant, this pattern indicated an upward trend in the number of shared photos or videos and comments received (between the first and second tertiles) when a recipe contained high sugar content, but the trend turned down when the recipe’s sugar level reached the third tertile. In terms of sugar, our data suggested that the number of comments was similar regardless of the fiber content. However, although not statistically significant, recipes containing the highest amount of fiber (third tertile) were less popular, having fewer shared photos and videos compared with those in the first or second tertile.

Multimedia Appendix 2 further presents the mean number of shared photos or videos and comments based on the recipes’ number of calories per serving. Data suggested that the number of shared photos and videos and the number of comments were positively correlated with the number of calories per serving. Recipes that provided more calories per serving (in the second and third tertiles) were more popular than recipes that provided fewer calories per serving (in the first tertile), with fewest shared photos or videos and comments.

### Comment Analysis

**Figure 2:**
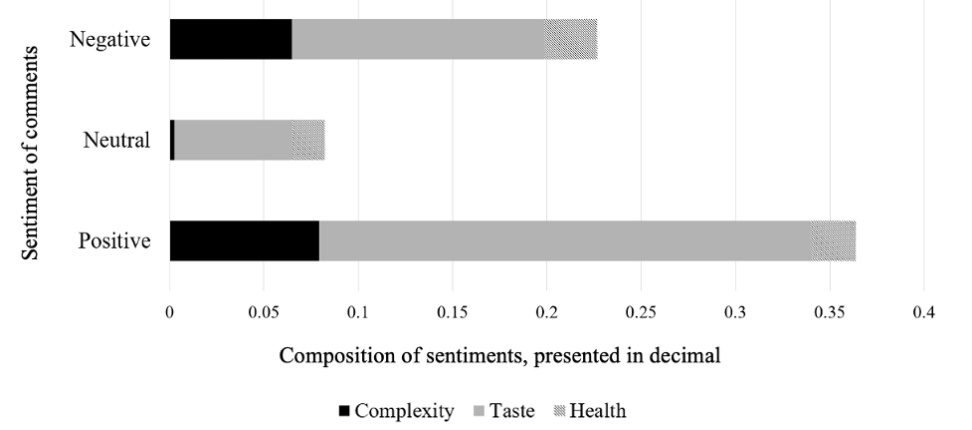
Pinterest users’ attitudes toward different aspects of recipes.

Table 2 shows sample comments, corresponding sentiment polarity, and related topic. Figure 2 shows the results of text mining from all 2818 comments analyzed. Out of 544 comments deemed as *taste* related, 25.9% (141/544) were positive, significantly higher than negative (72/544, 13.2%) and neutral (33/544, 6.1%; *P*<.05). The complexity of a recipe and its health attributes were commented on less frequently: less than 8% (225/2818) of the comments contained text related to complexity, and less than 3% (84/2828) of the comments contained text related to health. We found that *taste* and *complexity* were the most important factors in shaping Pinterest users’ sentiments.

**Table 2:** Comment samples and polarity.

Comment	Sentiment polarity	Keyword	Topic
“How long do I leave them in the oven?”	Neutral	N/A^a^	N/A
“How many calories is this?”	Neutral	Calories	Health
“I do not like brown sugar in my meatloaf...ugh”	Negative	N/A	N/A
“Definitely way too salty and too greasy for me.”	Negative	Salty and greasy	Taste
“It’s easy! I did this again and LOVED it!”	Positive	Easy	Complexity
“It turned out amazing!! Very delicious.”	Positive	Delicious	Taste

^a^N/A: not applicable.

## Discussion

### Principal Findings

In this study, recipes posted on Pinterest were collected, analyzed, and compared for ingredients and nutrients. We found that, in most cases, recipes using seafood or vegetables as the main ingredient, for example, tuna salad (main ingredient: tuna; other ingredients: celery, onion, flat-leaf parsley, mayonnaise, mustard, and black pepper), had, on average, fewer calories and less sodium, sugar, and cholesterol than meat- or poultry-based recipes, for example, crispy chicken wraps (main ingredient: popcorn chicken; other ingredients: tomatoes, cheddar cheese, buffalo wing sauce, and flour tortillas) and Mongolian beef (main ingredient: flank steak; other ingredients: cornstarch, canola oil, ginger, garlic, soy sauce, dark brown sugar, and scallions). Recipes using meat as the main ingredient, for example, creamy herbed pork chops (main ingredient: pork chops; other ingredients: milk, Montreal steak sauce, butter, flour, basil, black pepper, and instant beef bouillon granules), provided more energy by fat. Although the most followed pinners tended to post recipes containing more poultry or seafood and less meat, recipes serving higher fat or providing more calories per serving were more popular, having more shared photos or videos and comments. Sentiment analysis based on text mining showed that Pinterest users, in general, valued taste more than health qualities when making comments or sharing photos or videos.

With the sharp increase in the number of social media users, platforms such as Pinterest have become influential mechanisms to transform knowledge sharing and acquisition, including dietary choice [27]. According to a survey conducted by the Pew Research Center in 2018, about 28% of US adults used Pinterest [18]. Although evidence has shown that intervention tactics through tailored web-based platforms can help promote evidence-based nutrition education to the public [28,29], members of the academic community have urged for more research evaluating the mutual influence between social media users and information or content providers [30]. Our study provides the first glimpse into how recipe information is disseminated and viewed from 2 distinct perspectives: Pinterest posters (pinners) and users.

From the perspective of content providers, we found that the most popular pinners, by sharing recipes containing more seafood and poultry (Multimedia Appendix 1) with less sodium, sugar, and cholesterol, are overall more health conscious. This finding can be understood in terms of the social cognitive theory. The social cognitive theory states that a human is an agent that has not only been a forethinker but also a motivator and self-regulator. In a sense, humans learn by observing others’ actions and their consequences [31]. In our case, pinners are more likely to imitate posts that are socially rewarded. Early research on popular food blogs (webpages that can be pinned to Pinterest) aligned with our findings, suggesting that vegetarian and seafood recipes had significantly lower nutrition risks and more health benefits compared with red meat and poultry recipes [32]. Many health-conscious *social elites* do not eat meat at all; they only eat vegetables or seafood. It is not surprising that celebrities or social influencers, such as those with many followers on Pinterest, embrace this *elite social norm*, considering that most Pinterest users are from high-income households [18], which reinforced the role of self-reactiveness portrayed in the social cognitive theory [31]. In theory, an agent acts intentionally [31]. Popular pinners, such as celebrities, might be more health orientated and have implicitly or explicitly engaged in education, inspiration, and activism—the 3 stages of celebrity narratives—when they post recipes on Pinterest [33]. Red meat, for example, is classified as probably carcinogenic to humans (group 2A) by the Working Group of the International Agency for Research on Cancer [34] and is associated with type 2 diabetes, cardiovascular diseases, malignancies, and other diseases [35,36]. Such joint activity requires commitment to a shared intention [31,37]. Pinners may or may not be aware of this fact, but by posting recipes containing less red meat, they may have contributed to shifting public dietary choices to a healthier direction.

From the users’ perspective, they are often learners in pursuit of inspirational recipes [38]. We found that recipes with higher fat and sugar content tended to generate higher user engagement and greater numbers of shared photos or videos and comments. High engagement refers to actual offline use of the recipe rather than simply clicking *like* on a particular pin [24]. The social cognitive theory postulates that people are more motivated when they consider a subject worthwhile [37]. Our sentiment analysis corroborated the theory and literature by showing that users attached more importance to the taste of a recipe than its healthfulness and complexity. The social cognitive theory of mass communication also shows that behavior changes of an agent can be directly affected by media and indirectly influenced by connections to social systems that are diffused by media. The diffusion process relies heavily on the social-prompting power of the modeling [31]. Previous qualitative research showed that learners often quickly assumed the role of an expert or teacher when sharing nutrition information from social media [38]; therefore, it is concerning that ordinary people paid more attention to taste and were motivated by fat- and sugar-heavy pins. A likely downstream effect of these preferences of ordinary people is that *tasty* recipes will be disseminated quickly on social media through users’ social networks (a part of the social system) and make their way into regular recipe rotations for more people [39]. Our findings suggest a high priority area for future social media–based nutrition interventions.

There appears to be a discrepancy between what pinners posted and how users consumed information, leading to an opportunity for future health interventions via Pinterest. Previous studies have shown that social media interventions can have a positive effect on nutritional outcomes [14]. Strategies to increase users’ health consciousness can include, but are not limited to, (1) encouraging pinners to provide healthier (low sugar and cholesterol) alternative ingredients, (2) promoting recipes provided by health professionals and supported by evidence-based research [40,41], and (3) designing and promoting healthy recipes that are tasty and easy to prepare.

### Limitations

This study had some limitations. First, because of the restrictions imposed by Pinterest, the content scrolling process is not automated. The manual data collection resulted in a relatively small sample size and a large margin of error. To address the issues related to the small sample size, we applied a machine learning technique to mine text from the comments. A total of 100 comments were randomly selected to assess sentiment error rates. We found that the error rate was 18%, which is better than the acceptable level used in previous studies by convention [42]. Second, only recipes posted in English were included. Thus, the sample was not representative of non–English-speaking cultures or users. Third, the measurement of healthfulness was assessed based on the types of food ingredients and amount of fat, sugar, and fiber; other aspects of health, such as cooking methods, were not included. Future research should incorporate these aspects. Finally, demographic information such as gender or race and ethnicity of the Pinterest users was unavailable from Pinterest. Our sample was restricted to those who could adopt the food culture embedded in Pinterest. As seafood- or vegetable-only recipes are often more expensive or beyond the reach of low-income populations, more research is needed to address the potential socioeconomic disparities inherent in popular social media platforms.

### Conclusions

In this study, we used both content analysis and NLP techniques to analyze recipes posted on Pinterest. Seafood-based recipes and vegetarian recipes had fewer calories and less sodium, sugar, and cholesterol than meat-based recipes. Although the most popular pinners tended to exhibit more health consciousness by posting recipes with more seafood, poultry, and vegetables and less meat, recipes with higher fat and sugar content had higher user engagement, as demonstrated by the higher numbers of photo or video shares and comments. Population health could be improved with targeted interventions to address this disparity through efforts to enhance interest in and adoption of healthy recipes by Pinterest users.

## Appendix

Multimedia Appendix 1 Relationship between pinners’ popularity and their recipe ingredients.

Multimedia Appendix 2 Association between popularity and recipes.

## Abbreviations

NLP: natural language processing
VADER: valence aware dictionary and sentiment reasoner

## References

[1] Zarrinpar A, Chaix A, Panda S (2016). Daily eating patterns and their impact on health and disease. Trends Endocrinol Metab.

[2] St-Onge M, Ard J, Baskin ML, Chiuve SE, Johnson HM, Kris-Etherton P, Varady K, American Heart Association Obesity Committee of the Council on LifestyleCardiometabolic Health; Council on Cardiovascular Disease in the Young; Council on Clinical Cardiology;Stroke Council (2017). Meal timing and frequency: implications for cardiovascular disease prevention: a scientific statement from the American Heart Association. Circulation.

[3] Hutto CJ, Gilbert E (2015). VADER: a parsimonious rule-based model for sentiment analysis of social media text. Proceedings of the Eighth International AAAI Conference on Weblogs and Social Media.

[4] Kåhrström CT, Pariente N, Weiss U (2016). Intestinal microbiota in health and disease. Nature.

[5] Owen L, Corfe B (2017). The role of diet and nutrition on mental health and wellbeing. Proc Nutr Soc.

[6] Zhao Z, Yin Z, Zhao Q (2017). Red and processed meat consumption and gastric cancer risk: a systematic review and meta-analysis. Oncotarget.

[7] Damjanovska G, Severova G, Cakalaroski K, Antovska-Knight V, Danilovska I, Simovska V, Ivanovski N (2018). Beneficial short term effect of low protein diet on chronic kidney disease pro-gression in patients with chronic kidney disease stage G3a. A pilot study. Hippokratia.

[8] Hussain K, Murdin L, Schilder AG (2018). Restriction of salt, caffeine and alcohol intake for the treatment of Ménière's disease or syndrome. Cochrane Database Syst Rev.

[9] Yu E, Malik VS, Hu FB (2018). Reprint of: cardiovascular disease prevention by diet modification: JACC health promotion series. J Am Coll Cardiol.

[10] Bowman SA, Clemens JC, Friday JE, Lynch KL, LaComb RP, Moshfegh AJ (2017). Food patterns equivalents intakes by Americans: what we eat in America, NHANES 2003-2004 and 2013-2014. Food Surveys Research Group.

[11] Bowman SA, Clemens JC, Martin CL, Anand J, Steinfeldt LC, Moshfegh AJ (2017). Added sugars intake of Americans: what we eat in America, NHANES 2013-2014. Food Surveys Research Group.

[12] Lois S, Carrie LM, Joseph DG, Moshfegh AJ (2020). Meeting dietary guidelines recommendations: older adults what we eat in America, NHANES 2013-2016. Food Surveys Research Group.

[13] Coates AE, Hardman CA, Halford JC, Christiansen P, Boyland EJ (2019). Social media influencer marketing and children's food intake: a randomized trial. Pediatrics.

[14] Klassen KM, Douglass CH, Brennan L, Truby H, Lim MS (2018). Social media use for nutrition outcomes in young adults: a mixed-methods systematic review. Int J Behav Nutr Phys Act.

[15] Reicks M, Kocher M, Reeder J (2018). Impact of cooking and home food preparation interventions among adults: a systematic review (2011-2016). J Nutr Educ Behav.

[16] Adam M, Young-Wolff KC, Konar E, Winkleby M (2015). Massive open online nutrition and cooking course for improved eating behaviors and meal composition. Int J Behav Nutr Phys Act.

[17] Carlson N (2012). Inside Pinterest: an overnight success four years in the making.

[18] Perrin A, Anderson M Share of U.S. adults using social media, including Facebook, is mostly unchanged since 2018. Pew Research Center.

[19] (2016). Pinterest media consumption study.

[20] Chau MM, Burgermaster M, Mamykina L (2018). The use of social media in nutrition interventions for adolescents and young adults-a systematic review. Int J Med Inform.

[21] Guidry JP, Carlyle K, Messner M, Jin Y (2015). On pins and needles: how vaccines are portrayed on Pinterest. Vaccine.

[22] Simpson CC, Mazzeo SE (2017). Skinny is not enough: a content analysis of fitspiration on Pinterest. Health Commun.

[23] U.S. Department of Health and Human Services and U.S. Department of Agriculture (2015). 2015–2020 Dietary Guidelines for Americans. 8th Edition.

[24] Paige SR, Stellefson M, Chaney BH, Alber JM (2015). Pinterest as a resource for health information on chronic obstructive pulmonary disease (COPD): a social media content analysis. Am J Health Educ.

[25] Hutto C, Gilbert E (2014). VADER: a parsimonious rule-based model for sentiment analysis of social media text. Proceedings of the International AAAI Conference on Web and Social Media.

[26] Elbagir S, Yang J (2020). Sentiment analysis on Twitter with Python’s natural language toolkit and VADER sentiment analyzer. IAENG Transactions Eng Sci.

[27] Dumitrescu D (2016). Nonverbal communication in politics. Am Behav Sci.

[28] Gibson S, Adamski M, Blumfield M, Dart J, Murgia C, Volders E, Truby H (2020). Promoting evidence based nutrition education across the world in a competitive space: delivering a massive open online course. Nutrients.

[29] Blum ER, Stenfors T, Palmgren PJ (2020). Benefits of massive open online course participation: deductive thematic analysis. J Med Internet Res.

[30] Fung IC, Blankenship EB, Ahweyevu JO, Cooper LK, Duke CH, Carswell SL, Jackson AM, Jenkins JC, Duncan EA, Liang H, Fu K, Tse ZT (2020). Public health implications of image-based social media: a systematic review of Instagram, Pinterest, Tumblr, and Flickr. Perm J.

[31] Bandura A (2001). Social cognitive theory of mass communication. Media Psychol.

[32] Schneider EP, McGovern EE, Lynch CL, Brown LS (2013). Do food blogs serve as a source of nutritionally balanced recipes? An analysis of 6 popular food blogs. J Nutr Educ Behav.

[33] Beck CS, Aubuchon SM, McKenna TP, Ruhl S, Simmons N (2014). Blurring personal health and public priorities: an analysis of celebrity health narratives in the public sphere. Health Commun.

[34] Turesky RJ (2018). Mechanistic evidence for red meat and processed meat intake and cancer risk: a follow-up on the International Agency for Research on Cancer Evaluation of 2015. Chimia (Aarau).

[35] Wang X, Lin X, Ouyang YY, Liu J, Zhao G, Pan A, Hu FB (2016). Red and processed meat consumption and mortality: dose-response meta-analysis of prospective cohort studies. Public Health Nutr.

[36] Misra R, Balagopal P, Raj S, Patel TG (2018). Red meat consumption (Heme Iron Intake) and risk for diabetes and comorbidities?. Curr Diab Rep.

[37] Bandura A (2001). Social cognitive theory: an agentic perspective. Annu Rev Psychol.

[38] Adamski M, Truby H, M Klassen K, Cowan S, Gibson S (2020). Using the internet: nutrition information-seeking behaviours of lay people enrolled in a massive online nutrition course. Nutrients.

[39] Tobey LN, Mouzong C, Angulo JS, Bowman S, Manore MM (2019). How low-income mothers select and adapt recipes and implications for promoting healthy recipes online. Nutrients.

[40] Akhlaghi M, Ghasemi-Nasab M, Riasatian M (2020). Mediterranean diet for patients with non-alcoholic fatty liver disease, a systematic review and meta-analysis of observational and clinical investigations. J Diabetes Metab Disord.

[41] Abbasi J (2018). Interest in the ketogenic diet grows for weight loss and Type 2 diabetes. J Am Med Assoc.

[42] Roebuck K (2011). Sentiment Analysis: High-impact Strategies - What You Need to Know: Definitions, Adoptions, Impact, Benefits, Maturity, Vendors.

