# Supplementary figures and images for: Healthfulness Assessment of Recipes Shared on Pinterest: Natural Language Processing and Content Analysis
Source: J Med Internet Res. 2021 Apr 20;23(4):e25757. doi: 10.2196/25757 (PMC8097524; doi:10.2196/25757)

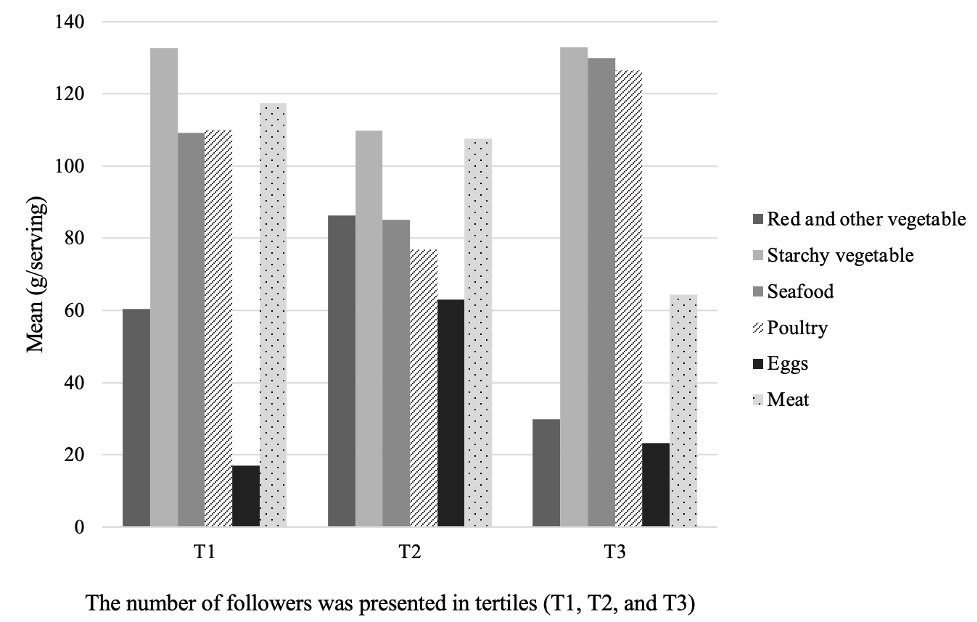

Supplement: Multimedia Appendix 1 [file jmir_v23i4e25757_app1.png]

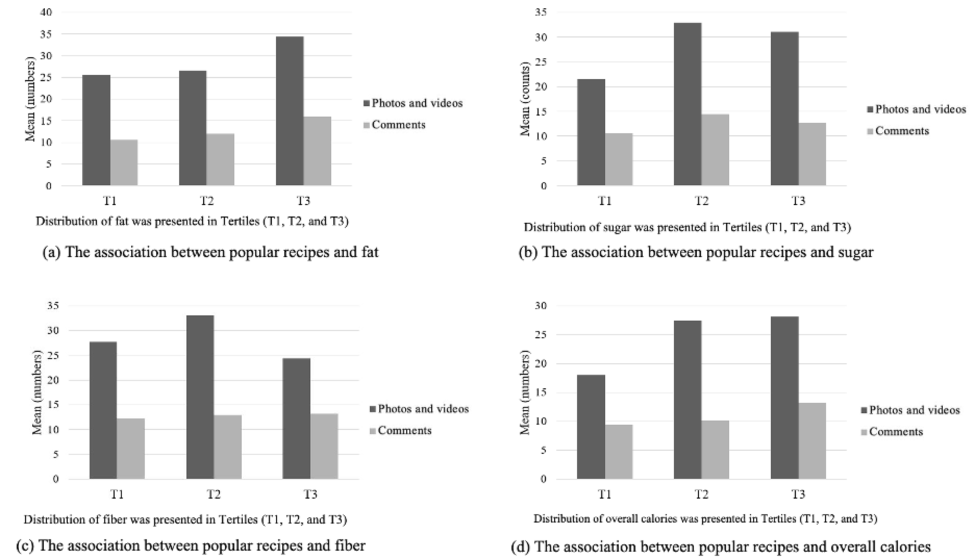

Supplement: Multimedia Appendix 2 [file jmir_v23i4e25757_app2.png]
